# Assistive Hearing Technology for Deaf and Hard-of-Hearing Spoken Language Learners

**DOI:** 10.3390/educsci9020153

**Published:** 2019-06-19

**Authors:** Rachael Frush Holt

**Affiliations:** Department of Speech and Hearing Science, The Ohio State University, Columbus OH, 43210, USA;

**Keywords:** digital hearing aids, cochlear implants, spoken language development, Biopsychosocial systems theory

## Abstract

Radical advancements in hearing technology in the last 30 years have offered some deaf and hard-of-hearing (DHH) children the adequate auditory access necessary to acquire spoken language with high-quality early intervention. However, meaningful achievement gaps in reading and spoken language persist despite the engineering marvel of modern hearing aids and cochlear implants. Moreover, there is enormous unexplained variability in spoken language and literacy outcomes. Aspects of signal processing in both hearing aids and cochlear implants are discussed as they relate to spoken language outcomes in preschool and school-age children. In suggesting areas for future research, a case is made for not only expanding the search for mechanisms of influence on outcomes outside of traditional device- and child-related factors, but also for framing the search within Biopsychosocial systems theories. This theoretical approach incorporates systems of risk factors across many levels, as well as the bidirectional and complex ways in which factors influence each other. The combination of sophisticated hearing technology and a fuller understanding of the complex environmental and biological factors that shape development will help maximize spoken language outcomes in DHH children and contribute to laying the groundwork for successful literacy and academic development.

## 1. Introduction

Assistive hearing technology for deaf and hard-of-hearing (DHH) children has seen great advancements in the last 50 years. Certainly, there are educators nearing retirement who remember when young DHH children regularly wore body aids and there are mid-career educators who remember when DHH children wore large, analog, linear hearing aids as the standard, or even just one hearing aid, despite having bilateral hearing loss. Today, there are no children in US classrooms wearing body aids and very few wearing analog, linear hearing aids that dangle below their earlobes. Technology has come a long way not only in form, but also in function. While form certainly matters (many children like the flexibility of being able to add stickers to their devices, wear hearing aids that are the same size as or even smaller than their ears, and select multi-colored earmolds—so do some adults!), radical advances in hearing technology has opened the door to the possibility of spoken language development for many (but not all) DHH children whose parents desire it.

This review focuses on current-day hearing technology for children with permanent hearing loss, also known as sensorineural hearing loss. Typically, this type of hearing loss is due to damage to the cochlea but can also include damage to the auditory nerve and, occasionally, structures in the central auditory system. Sensorineural hearing loss not only results in reduced audibility, but also reduced spectral resolution (frequency or “pitch” is not heard as clearly as it is with typical hearing), poor temporal processing (difficulty following changes in sound that occur over time), reduced binaural abilities (using both ears in tandem for locating where a sound originates in space and listening effectively in background noise), and loudness recruitment (an abnormally fast growth in loudness—the perception of intensity—of sound) [1]. The combined effects of sensorineural hearing loss on speech perception are far reaching. First, not all speech sounds (or phonemes) are equally audible. Soft sounds, such as the “th” in the word “thawed,” will likely be inaudible. Depending on the degree (mild to profound loss) and configuration (shape of the loss—flat or sloping) of the hearing loss, some moderate-level sounds (such as the “d” in “thawed”) could be inaudible as well. Louder sounds, such as the “aw” in “thawed,” could be audible, but for those with more severe losses, will also not be audible. Very loud sounds will reach uncomfortable levels quickly, further reducing the range over which listeners have usable hearing (their dynamic range). Furthermore, distortion caused by reduced spectral resolution and temporal processing can cause even audible sounds to be difficult to understand and loud sounds can be further distorted. Reduced audibility and binaural abilities and poor temporal and pitch processing can all cause difficulty listening in background noise. Hearing technology is very good at addressing reduced audibility and is an “engineering marvel” [1] (p. 207). However, reduced dynamic range (particularly in the case of listeners with severe-to-profound hearing loss), and temporal and spectral resolution are some of the biggest challenges in successfully fitting listeners with hearing technology. The remaining challenge, or what many refer to as the “holy grail” [2] (p. 36), is addressing listeners’ difficulty hearing in background noise.

The two primary types of hearing technology used by school-age children are hearing aids and cochlear implants. Hearing aids and cochlear implants each take fundamentally different approaches to delivering acoustic signals to the listener. Hearing aids amplify signals and transmit them to the listener’s ear canal, whereas cochlear implants convert the acoustic signal into pulses of electrical current that are emitted directly into the listener’s organ of hearing—their cochlea. The electrical pulses stimulate neural units from the auditory nerve along a portion of the cochlea. Those neural units then propagate information from the signal up the auditory pathway to the auditory cortex in the brain. The reason cochlear implants directly stimulate auditory nerve fibers is because they are intended to bypass irreparably damaged structures in the cochlea of listeners with severe-to-profound sensorineural hearing loss. In other words, cochlear implants are intended for listeners who do not benefit from hearing aids.

## 2. Hearing Aids

There are many ways to classify hearing aids: by where they are worn on the body, by the type of amplifier they use, by the type of signal processing they employ, by their size, or even by a combination of these factors. For modern hearing aids, most are worn on or in the ear. There are implantable styles of hearing aids but many of them are not yet Food and Drug Administration (FDA)-approved for widespread use in young children, so we will concern ourselves with hearing aids that are worn on or in the ear, because they are the most commonly encountered ones today with children. Hearing aids that are custom fit to sit inside the ear canal or in the “bowl” of the ear around the entrance of the ear canal are not typically worn by children. There are several reasons for this: (1) children’s ears are constantly growing, particularly quickly when they are infants and toddlers, through to 8–10 years of age [3]. This style of custom hearing aid would require constantly re-casing the hearing aid to keep up with ear growth, which is impractical. (2) Often times these styles of hearing aids cannot couple with digital modulation (DM) systems used in classrooms. Therefore, far and away the most common style of hearing aid used by school-age children, toddlers, and infants are behind-the-ear (BTE) hearing aids [4]. BTE hearing aids include the standard style, which contains an ear hook that connects the hearing aid to an earmold that delivers the amplified signal to the ear canal. The ear hook sits on top of the ear and allows the hearing aid to hang behind the ear (hence the name of the device). This style of BTE is the most flexible and durable hearing aid on the market. Another style of BTE moves the receiver from the casing behind the ear to inside the ear/ear canal (RITE/RIC). RITEs/RICs use the same type of BTE casing that sits behind the ear, but rather than the traditional tubing used on standard BTEs, they use a thin “wire” (plastic, not metal) that connects to a flexible plastic “dome.” Domes are offered in various sizes depending on the degree of hearing loss and size of the ear canal. The less severe the hearing loss, the smaller the dome and the more porous the dome can be. RITE/RIC technology grew out of the confluence of noise suppression technology and the ability to miniaturize hearing technology [5]. The result is hearing technology that minimally occludes the ear canal without feedback (that squeal that can be heard when an amplified acoustic signal is fed back into the microphone). BTEs as a group vary in size, but in general are the largest of the ear-level devices (hearing aids that are worn on or in the ear), thereby making them quite durable, usually with larger and thus longer-lasting batteries than other styles that use smaller batteries.

As mentioned earlier, most children are fit with standard BTEs [4]. Some audiologists have been fitting RITEs/RICs on children after they found success fitting them on adults, and manufacturers have recently marketed RITEs/RICs for children. However, few peer-reviewed studies have been carried out with these devices to evaluate their efficacy in children. Concerns that have been raised about their use with children include [5]: they may not include a telecoil or direct audio input, which allows them to couple to audio and assistive listening devices; they may not be compatible with DM systems in the classroom; the cost of replacing the coupling system as the child grows (particularly RITE receivers) can be significant; a lack of durability for some devices can be of great concern with children who tend to be hard on technology; smaller battery size means a shorter battery life; the potential for transient middle-ear problems is great in the pediatric population, as is progressive hearing loss for some children, and the open fit combined with the gain algorithms used do not provide amplification below 1000 Hz—combined, this could result in inadequate amplification for children in the mid-to low-frequencies. Finally, probe mic measurements are vital with RITEs/RICs in children because fitting ranges for open-fit hearing aids are based on measurements from couplers (hard-walled closed cavities), which do not approximate gain well, particularly in the low frequencies. In summary, more research is needed on the efficacy of RITEs/RICs in the pediatric population.

BTEs (or any style of hearing aid, for that matter) employ an amplifier (the component that increases the strength of the acoustic signal). Amplifiers are either linear or nonlinear. At any given frequency, a linear amplifier amplifies an acoustic input by the same amount, regardless of the level of the input or what other sounds are present; a nonlinear amplifier varies the amount of amplification depending on the level of the input signal. Finally, most hearing aids use digital signal processing (much like a CD player or iPod) and many utilize wireless technology for certain applications. Digital signal processing takes a continuous electrical signal (an analog signal) and converts it to numerical values that occur at discrete moments in time. The virtue of this processing is that it allows the development and application of algorithms that can do mathematical manipulations of those numbers in ways that cannot be done with analog processing. In this way, digital signal processing has opened the door to many sophisticated processing algorithms for addressing problems that listeners with sensorineural hearing loss experience, such as problems listening in background noise.

### 2.1. Basic Components of Hearing Aids

All hearing aids, regardless of type, contain the same basic parts:

- A *microphone* or multiple microphones to convert the acoustic signal into an electrical signal
- An *amplifier* to differentially increase the power of the electrical signal across frequencies
- A *receiver,* which is like a small loudspeaker, to convert the electrical signal back into an acoustic signal
- A *battery* to provide power to the amplifier

### 2.2. Signal Processing in Hearing Aids and Spoken Language Outcomes in School-Age Children

Likely, the area of hearing aid design of most interest to educators relates to how hearing aids address background noise. Background noise consists of any deleterious sound that interferes with the ability to hear and understand the signal of interest. Most background noise sources include heating, ventilation, and air conditioning (HVAC) systems, fluorescent lights, other children in the classroom, next-door classrooms and hallways, noise from the outdoors (cars, airplanes, playground, etc.), chairs and desks sliding on the floors, and classroom pets. Background noise negatively affects spoken language processing (e.g., [6-8]) and academic achievement [9,10] in school-age children by interfering with the signal of interest, which is often the teacher in a classroom setting but can also be the student’s classmates during classroom discussion or other peer interactions. Unfortunately, classroom noise levels vary from −7 to +10 dB SNR across studies [11,12], despite the recommended level being +15 to +30 dB SNR [13]. SNR stands for “signal-to-noise ratio,” which is a misnomer, because it is not a ratio, it is a difference value. SNR refers to the difference in level between a signal of interest and the ambient noise in the environment. In this case, it refers to the average difference in level between the teacher’s voice and the ambient noise in the classroom environment. If the SNR is +2 dB, it means the teacher’s voice is 2 dB more intense than the noise in the classroom, if the SNR is 0 dB, it means the teacher’s voice and the noise are equal in level, and if the SNR is −2 dB, it means the teacher’s voice is 2 dB less intense than the noise in the classroom. Based on the data from these classroom noise studies, even the most acoustically friendly classrooms still have background noise levels that are 5 dB too high for ideal learning. These noise levels are detrimental for spoken language processing and academic success for all students. However, they are particularly problematic for children with hearing loss who are already at risk for missing auditory information. Further, children are not the only ones experiencing difficulty in typical American classrooms. Teacher vocal fatigue can be a byproduct of having to overcome the high noise levels in classrooms, which can lead to vocal nodules and other overuse injuries to the vocal folds. Current hearing aids address background noise in two different ways. The first is by using directional microphones and the second is by using digital noise reduction technology.

## 3. Directional Microphones

In BTE hearing aids the microphone(s) sits on the plastic case just above the ear, collects acoustic signals, and converts them into electrical signals. Hearing aid microphones can be omnidirectional, meaning that they collect signals equally 360 degrees in a plane around them, or directional, meaning that they suppress signals coming from a specific direction (typically from behind the listener). The reason directionality might be employed is based on the assumption that the majority of the auditory signals of interest are thought to originate from in front of the listener (e.g., a friend talking with a child or a teacher giving a math lesson, etc.). To the degree that this is in fact true, reducing the amount of sound coming from behind the listener (e.g., background noise that could interfere with the signal of interest) that gets processed by the hearing aid from the outset could provide an enhanced signal for the listener. Directional microphones improve the SNR by 2–3 dB [14]. However, for directional microphones to provide this small increase in audibility, the child either needs to orient themselves toward the signal of interest regularly and/or their automatic directional algorithm needs to reliably detect from where the signal of interest originates and respond appropriately. Ching et al. [15] reported that both hearing and DHH 1- to 6-year-olds only look at the talker approximately 40% of the time. However, recent findings suggest that despite rarely looking at the talker (using eye-tracking), school-age children are able to follow auditory instructions in a behavioral task, with hearing children outperforming DHH children [16]. In sum, children look at the signal of interest less than half of the time, including those with hearing loss [15-17]. These results suggest that children with hearing aids will not naturally benefit from directionality a good portion of the time. However, directional microphones have not been demonstrated to negatively impact speech perception and thus, are routinely recommended for pediatric hearing aid fittings [4,18].

## 4. Digital Noise Reduction

Digital noise reduction encompasses a wide array of signal processing strategies intended to categorize what components of a signal are speech and what are noise, and to reduce the amount of gain the hearing aid provides when it detects primarily noise. The vast majority of research on digital noise reduction to date has been completed on adults. Those data suggest that digital noise reduction does not improve listeners’ spoken word recognition, nor does it hamper it, rather, it makes the listening experience more pleasant under high levels of noise (e.g., [19]). Similarly, in the few investigations that have been carried out with DHH children, digital noise reduction did not enhance nor hinder spoken word recognition [20], nor children’s ability to learn novel words [21]. Therefore, digital noise reduction requires further research before strong conclusions can be made regarding its impact on spoken language development in children.

Perhaps the most promising work to date to address listening in noise has been carried out by Healy and Wang and their colleagues. They have developed a novel algorithm to cleverly segregate the speech signal of interest from background interfering speech (which is often the source of background noise) that has demonstrated significantly improved speech intelligibility for adults in laboratory settings (e.g., [22]). Tests of this algorithm on ear-level devices (e.g., hearing aids and cochlear implants) are forthcoming. It will be exciting to test this algorithm with children at some point in the future.

## 5. Prescribing Gain: The Importance of Audibility

The primary goal of hearing aids is to restore audibility to DHH listeners. For the purpose of developing spoken language, restoring audibility is often described in reference to the speech signal: the goal is to make the long-term average spectrum of speech (LTASS) audible. Full access to the speech signal is the first of many steps in providing children the necessary input required to begin developing spoken language [23]. Prescriptive fitting rules that are used for calculating how much gain to prescribe by the amplifier at each frequency are concerned with making the LTASS audible, but not uncomfortably loud. In laboratory settings, there tends to be little difference in average speech intelligibility, language, and speech production across the available fitting rules that are used explicitly with children (e.g., [24]). However, reports in real-world clinical settings suggest that less than half of children’s hearing aids are fit such that at least one ear is within +/− 5 dB of their prescriptive gain targets and that the rate of poor match-to-target increases at higher frequencies (e.g., [25,26]). These findings hold true regardless of the degree of hearing loss. Results such as these suggest that there is important work to be done in fitting and verification of pediatric amplification. If children do not have access to the signal of interest—speech—it clearly makes the task of learning spoken language exponentially harder. Once the hearing aid fit is verified in the clinical setting, validating it in the classroom setting not only ensures that the child’s hearing aid meets their everyday needs in their learning environment(s), it also confirms for both their caregiver(s) and teacher(s) and that they have adequate access to the LTASS, and maximizes the partnership between audiologists, families, and school personnel. A word of caution that brings us back to the beginning of this section on audibility: full access to the LTASS is the first of many steps in achieving spoken language competency and academic growth; it is a necessary first step, but much more therapy and education is required.

Stelmachowicz and colleagues’ work, as well as that of many other research teams, has been formative in our understanding of the role of speech audibility—the primary purpose of amplification—in spoken language development. All listeners need access to some degree of an audible signal in order to understand a speaker. However, children need a more audible signal—or a greater portion of the LTASS to be audible—than do adults, in order to maximize their spoken word recognition [27]. This is because children have less experience with spoken language and the world more generally than adults, and are less facile with filling in missing or missed linguistic information. Furthermore, adequate access to high-frequency speech information is particularly informative for both speech perception [28] and spoken language production [29]. Hearing aids do not amplify signals above approximately 5 kHz, due primarily to technical limitations. This has a consequence for perception, but also impacts the ability to self-monitor one’s own speech. For example, Elfenbein, Hardin-Jones, and Davis [30] demonstrated that even children with mild hearing losses misarticulate and/or omit high-frequency fricatives. Their findings support the view that DHH children might not have access to the high-frequency cues necessary to monitor one’s own speech that are required to develop a full phonological inventory.

Phonological development is markedly delayed in DHH children relative to hearing peers [29,31]. Delays tend to correlate with the relative audibility of phonemes across the frequency spectrum with delays being shortest for vowels (primarily low-frequency concentration) and greatest for fricatives (primarily high-frequency concentration). Unfortunately, roughly half of the consonants used in spoken English are fricatives [29], therefore, delays in acquiring them can have substantial effects on spoken language development. Moeller et al.’s [31] data suggest that fricative acquisition is strikingly delayed in DHH children relative to hearing children, even in DHH children who were fit with amplification before six months of age. Other speech sound classes were delayed as well, but the rate of acquisition of those classes were similar to hearing children. Supporting the importance of audibility, Moeller et al. [31] argued that the lack of access to high-frequency amplification due to limitations of the devices themselves were the primary underlying cause of children’s marked delay in fricative acquisition. Fricatives are not only important because they are used so often in running speech, some of them, such as /s/ and /z/, also are morphological markers for items such as plurals and possessives, making them important for the acquisition of morphosyntax [32].

## 6. Frequency Lowering

A critical reader might ask, if high frequencies are so important for perception, why don’t hearing aid manufacturers increase the bandwidth of hearing aids beyond 5000 Hz? The simple answer is that there are technical limitations, an increased susceptibility to acoustic feedback, and limits on output that have restricted the ability of engineers to widen hearing aid bandwidths. A clever end-around to this problem, called “frequency lowering,” has been implemented in modern hearing aid technology. There are multiple approaches to frequency lowering, but generally, frequency lowering takes high-frequency acoustic information that is not typically accessible to the listener and spectrally lowers it down to a region that is audible. It has been estimated that frequency lowering is used on upwards of 80% of a common manufacturer’s pediatric fittings [33]. Despite this, there are few studies that have carefully examined the effects of frequency lowering on spoken language in DHH children. Whereas these studies report advantages for frequency lowering, they have some limitations. Across these seminal investigations, the fitting scheme and the type of frequency lowering varied, making comparisons across studies difficult (e.g., [34-36]), or evaluated outcomes based on aided pure-tone averages (e.g., [35]), which is limited for predicting speech audibility under typical listening conditions because thresholds are obtained for input levels measurably below the average input level for conversational speech [37]. Furthermore, studies that examined spoken language did not include control conditions that address practice effects and maturation (e.g., [38]). Despite this, in their review of frequency lowering technology for children, McCreery, Venediktov, Coleman, and Leech [39] argued that many studies found that children reported that they preferred frequency lowering technology over conventional frequency mapping. These early results of the effects of frequency lowering in children are somewhat promising and offer the opportunity for further research in this area to determine who is likely to benefit the most from it. Recent work by Scollie et al. [40] have attempted to develop guidelines for verifying fit of frequency lowering technology that maximizes the contrasts between high-frequency fricatives. Future work on this approach with children is warranted.

## 7. Amplitude Compression

As discussed earlier, one of the consequences of sensorineural hearing loss is reduced dynamic range—or the ranges of intensities over which one is able to hear. A healthy ear has a dynamic range of approximately 120 dB, while the dynamic range of speech of an individual talker is about 30 dB. Across talkers, the dynamic range of speech is upwards of 60 dB. As sensorineural hearing loss sets in, soft sounds become inaudible and audible sounds reach maximum comfort levels faster than normal, and the dynamic range of hearing can start to encroach upon the dynamic range of the speech. In the case of severe-to-profound hearing loss, the dynamic range of speech can eclipse the dynamic range of hearing. In this case, the audiologist faces a dilemma: amplify soft sounds so that they are audible, while making loud sounds uncomfortably or painfully loud (resulting in the user not wearing the hearing aids), or amplify loud sounds to the point just below discomfort while sacrificing the ability to hear soft sounds (resulting in the user not having access to the full LTASS, and thus having less-than-ideal spoken word recognition)? This was the dilemma that audiologists faced when fitting linear hearing aids. Today, all modern hearing aids use a technology called “amplitude compression.” Amplitude compression is a non-linear approach to signal processing in which soft sounds are provided more amplification or gain than moderate-level sounds and certainly than loud sounds. Theoretically, this allows the audiologist the ability to give the listener access to the entire LTASS, while also making sure that all sounds are maintained at a comfortable volume. Because compression alters the original signal [41], it is reasonable to question if it has an effect on spoken language development. In a review of amplitude compression versus conventional linear amplification for pediatrics by McCreery, Venediktov, Coleman, and Leech [42], over 376 potential papers were winnowed down to just eight that met the stringent inclusion criteria. There was some variability in spoken word recognition across the investigations for different presentation levels (e.g., low, medium, and high), with some studies finding better results for devices that used compression over linear processing for low and high levels, and more mixed results for medium-level inputs. Few studies investigated speech production, but that which did revealed improved articulation with the use of amplitude compression over linear processing [43]. McCreery et al. [42] concluded that there is a moderate level of evidence to support the use of amplitude compression over conventional linear processing for school-age children using hearing aids in certain areas of audibility, spoken word recognition, and speech and language development.

## 8. Summary of Spoken Language Outcomes in DHH Children with Hearing Aids

Hearing aid technology has changed dramatically in the last 30 years. Whereas this has made it feasible for many current DHH children to attain spoken language and academic achievements greater than the previous generation of DHH children [44], there remains an achievement gap between the majority of DHH children and their hearing peers who are matched on chronological age and socioeconomic status (e.g., [24,45,46]). There are occasional findings of null results in the literature regarding language differences between hearing children and children with mild to severe hearing loss who wear hearing aids (e.g., [47]), but the vast majority of investigations report different developmental patterns in spoken language development (perception and production) in DHH children with hearing aids from differences in phonological skills (e.g., [29,31,48]), morphosyntactic skills (e.g., [46]), vocabulary and grammar development [30,44,49], and spoken word recognition (e.g., [27]). Despite these delays and differences, longitudinal data from the Outcomes of Children with Hearing Loss (OCHL) study suggest that the auditory access provided by hearing aids, specifically those that are well fit and worn consistently, are absolutely critical for the development of spoken language (e.g., [46,50]).

## 9. Cochlear Implants

For children (and adults) whose degree of hearing loss is so severe that they do not benefit from hearing aids, cochlear implants offer an opportunity to receive access to sound. The phrase, “access to sound” was used intentionally because the development of spoken language does not generally occur automatically in children who receive cochlear implants. It is the product of years of aural (re-)habilitation, speech-language therapy, family dedication, and hard work on the part of the child, the family, and many professionals, including educators. Whereas aural (re-)habilitation should be a part of intervention for all DHH children, including those with hearing aids, aural (re-)habilitation is particularly critical for children with cochlear implants. They generally have sensorineural hearing loss that is severe-to-profound in degree, resulting in a period of time that they have not had adequate access to conversational speech, even that which is amplified by a hearing aid. Furthermore, the cochlear implant provides an entirely different signal than hearing aids. Thus, learning to listen to a new signal through an impaired auditory system generally requires an amount of training, effort, and time.

### 9.1. Cochlear Implant Candidacy through Surgery

Cochlear implant candidacy criteria are always evolving, are manufacturer-specific, and vary with candidate age, particular device, and whether the candidate has private insurance or is using Medicare. In general, the current pediatric audiological criteria for implantation according to the US Food and Drug Administration are as follows: 12- to 23-month-old infants must have bilateral, profound, sensorineural hearing loss and display little-to-no evidence of auditory development with appropriately fitted hearing aids; children ages two years and older must meet the same requirements except that their hearing loss can be severe in degree. For children who are too young to be tested with formal spoken word recognition tests, parental questionnaires that pose questions about auditory development are used. There is some evidence that infants implanted before 12 months of age develop better spoken language, but not better speech perception, than those implanted after 12 months of age (e.g., [45,51-54]). Furthermore, spoken language outcomes of children with cochlear implants are similar to those of children with hearing aids who have pure-tone averages in the moderately-severe range (e.g., [55,56]). Both of these lines of research suggest that the current FDA-approved age and audiometric criteria might be too stringent. That being said, most clinics use the guidelines described above to determine who qualifies for cochlear implantation.

Depending on the age of the child, candidacy for cochlear implantation is determined with a team approach that at minimum typically includes audiology, speech-language pathology, and otolaryngology. Oftentimes, large teams also include developmental psychology, social work, and other professionals. From an audiological standpoint, the child will undergo a large battery of behavioral and physiological testing to confirm the degree, type, and configuration of hearing loss, a thorough hearing aid trial with hearing aids in which the fit has been verified, and a large, hierarchical battery of spoken language tests called the Pediatric Minimum Speech Test Battery [57,58]. Speech-language pathology will provide a communication and spoken language assessment. All of these assessments not only help determine candidacy, they also help determine baseline performance in children who eventually go on for cochlear implantation. The surgeon will do a head and neck examination to look for otitis media and congenital anomalies, take a thorough family and medical history, check overall health, order imaging (Computerized Tomography scan [CT scan] or Magnetic Resonance Imaging [MRI]), and occasionally order vestibular (balance) testing. Once candidacy is determined, the family is provided with a wealth of information to help decide if they want to proceed with surgery.

Surgery is usually an outpatient procedure, lasting 2–3 h. It is minimally invasive, using a small, curved incision just behind the ear. Before the surgeon completes the procedure, most centers will confirm that the device is working by checking the integrity of the implanted electrodes and verifying the stimulation of the auditory nerve. Many surgeons will also obtain an X-ray or fluoroscopy of the internal device once it has been placed to ensure that it is indeed located where it is supposed to be inside of the cochlea. Cochlear implants themselves contain two major portions—an internal and an external portion. Only the internal portion is implanted inside the recipient’s head.

### 9.2. The Cochlear Implant

#### 9.2.1. Internal Components

The internal device of the cochlear implant looks different across manufacturers and across a single manufacturer’s different models, but they all contain the same basic components:

- A *magnet* that helps keep the external device on the user’s head
- An *internal receiver* that receives radio frequency waves from the external component’s transmitter and converts it into electrical energy
- A flexible *electrode array* containing between 12–24 intracochlear *electrodes,* which deliver electrical pulses to auditory nerve fibers within the cochlea that are in close proximity to each electrode

#### 9.2.2. External Components

The external device of the cochlear implant (often called the speech processor or sound processor) also looks different across manufacturers, as well as across the different types of processors within a single manufacturer. However, they all contain the same basic components:

- A *microphone* to convert acoustic signals into an electrical signals
- A *sound processor* that processes the electrical signal based on some logic regarding speech sound processing
- A *transmitter* that sends the signal across the skin on the head via radio frequency waves to the surgically implanted portion of the device
- A *magnet* that helps maintain the external device on the user’s head

## 10. How It Works: Device Basics

Cochlear implants work very differently from hearing aids. Whereas there is variability across manufacturers and across processing strategies regarding the specifics of how cochlear implants process acoustic signals and stimulate auditory nerve fibers, all cochlear implants do the same basic processing. After converting acoustic sound waves into electrical energy at the microphone, they all filter the electrical signal into contiguous frequency bands, amplitude compress the frequency bands, extract the envelopes of the filters, and modulate pulses from each electrode based on the extracted envelope. Importantly, the electrodes are located in physically distinct locations along the cochlea. Beginning at the cochlea and going all the way to the auditory cortex, the auditory system is organized in a “tonotopic” fashion, meaning that certain anatomical structures respond maximally to certain frequencies [1]. In the cochlea, which is shaped like a snail shell with 2.5 turns, the first turn is maximally sensitive to high (treble) frequencies and the last turn is maximally sensitive to low (base) frequencies. The cochlear implant electrode array is threaded into the first 1 to 1.5 turns of the cochlea (or 20–30 mm into the cochlea), such that low-frequency neural units are not necessarily directly stimulated. The entire purpose of modern-day cochlear implants having multiple electrodes inside of the cochlea is to stimulate distinct populations of neural units that are maximally sensitive to different frequencies within the cochlea. The intent is to provide the listener with the opportunity to perceive some frequency cues from the speech signal. Without electrodes placed in physically distinct locations along the cochlea, there would be no opportunity for the listener to perceive frequency cues, and it is known that perceiving frequency cues is important for speech perception (e.g., [59]).

Cochlear implants vary in how well they convey different cues in the speech spectrum to the listener. In general, they tend to be best at conveying speech cues that are conveyed well by temporal envelope cues, and are poorest at those that rely heavily on conveying fine frequency cues. The sounds that make up words—phonemes—can be classified into: manner of articulation, voicing, and place of articulation. Manner of articulation relies heavily on temporal envelope cues, whereas place of articulation (where in the mouth a sound is uttered) relies only on fine frequency cues [59]. From a functional standpoint, cochlear implant recipients generally can perceive manner of articulation relatively well, followed by voicing (are the vocal folds used or not to utter the sound), and are poorest at perceiving place of articulation. This means that they are going to me more likely to confuse the words “top” and “cop” than the words “mom” and “bomb,” because the first phonemes of “top” and “cop” only vary in place of articulation, whereas in “mom” and “bomb,” the first phonemes vary in manner of articulation.

### 10.1. Post-Operative Procedures: The MAP

For the first week following surgery, patients are instructed to undergo minimal activity. After 2–3 weeks, they visit the surgeon for a post-operative check to see how the incision site is healing and to evaluate general recovery. Approximately one month following surgery they visit the audiologist to receive the external portion of the device—this will be the first time they receive stimulation from the cochlear implant that allows them to perceive sound. One major reason for the delay is that the surgical site needs to heal and swelling needs to recede before the external magnet is placed on the head. During the visit in which they receive the external portion, the electrodes are “mapped,” meaning that the appropriate amount of current for each electrode is determined by the audiologist. This is a difficult process and one that is done both behaviorally and using physiological measurements. This will not be the child’s final MAP (a term that is used to describe the levels set on each electrode as a whole). The amount of current required to detect sound decreases over time, which will impact children’s MAPs [60,61]. MAPs are evaluated, modified, and adjusted at every single visit to the audiologist. Because current level needs are changing frequently in children, they are seen often early on for MAP evaluations. Input from parents, teachers, speech-language pathologists, and other individuals who know the child well can be useful in setting MAPs, particularly in preverbal children. Simple tests, such as the Ling 6-sound test [62], can be used to check whether the child can detect phonemes across the speech spectrum and includes the phonemes, “ee,” “oo,” “ah,” “s,” “sh,” and “m.” As with hearing aids, the goal is to make speech audible and comfortable, and to make auditory signals as clear as possible. However, whereas cochlear implants are arguably the most successful sensory prosthesis developed to date, they still produce a rather crude representation of sound.

Cochlear implant processing has improved markedly since the first single-channel device was implanted in a child in the United States by William House, M.D. in 1980. All modern cochlear implants are multichannel devices. Current cochlear implants offer multiple types of processing strategies, which can provide differences in perception for individual users, but on average the processing strategies result in similar outcomes across cochlear implant recipients (e.g., [63]). Regardless of the processing strategy, the signal provided by a cochlear implant lacks the spectral/frequency detail of the original signal—the utterance originating from the child’s mother’s mouth, the song being played by an orchestra or CD player, or the television broadcasting the child’s favorite program. Moreover, the signal sent from the electrodes is processed through an auditory system that is significantly impaired. Fortunately, we do not hear with our ears, rather, we hear with our brains. As one of the cochlear implant (CI) signal processing pioneers, Dr. Blake Wilson, ruminated, “in retrospect, the job of designers of CIs was to present just enough information in a clear format at the periphery such that brain could ‘take over’ and do the rest of the job in perceiving speech and other sounds with adequate accuracy and fidelity...The brain ‘saved us’ in producing the wonderful outcomes provided by the present-day CIs” [64] (p. 53). More simply stated by Dr. David Pisoni and the research team at the Indiana University School of Medicine, “the ear is connected to the brain” [65] (p. 446). Indeed, the fact that so many DHH children are able to make use of the degraded signal provided by cochlear implants for the development of spoken language suggests an amount of neural plasticity never imagined by those working with young DHH children early on in cochlear implant development.

### 10.2. Spoken Language Outcomes in Pediatric Cochlear Implantation

Spoken language outcomes in pediatric cochlear implant recipients are as variable as they can possibly be. For example, receptive and expressive language scores range from floor to ceiling in one of the largest studies to date of 188 children who received cochlear implants before age five years (e.g., [66]). These results are representative of most studies. Device characteristics, such as the number of active electrodes and the size of the dynamic range [67], and the number of distinct frequency channels [68], only account for a small fraction of the variability in outcomes. Whereas adult cochlear implant users are believed to only need approximately four spectral channels of information to perform maximally on spoken word recognition in quiet [69], young children need approximately eight frequency channels of information while listening in quiet settings (e.g., [68]), with more required in ambient noise to optimize their understanding of speech.

Factors that account for the variability in outcomes are nearly as diverse as the outcomes themselves. The most studied include: age at cochlear implantation, which also can be thought of as the length of auditory deprivation in many cases—in general, earlier implantation results in better language outcomes (e.g., [51,52,70,71]); degree of hearing loss prior to surgery—in general, those with more residual hearing see better spoken language and speech perception outcomes [66,72,73]; the family’s choice of communication modality—admittedly a difficult area to study because of confounding factors, but in general, oral approaches result in better spoken language and speech perception outcomes (e.g., [52,67,74-78]); the family’s role in therapy—in general, children from families who are actively engaged in the intervention process have better spoken language outcomes (e.g., [79,80]); socio-economic status—in general, children from families with more resources have better language outcomes, much like their hearing peers (e.g., [52,66,67,81]); ethnic minority status—one of the only studies to examine pediatric cochlear implant users who are ethnic minorities found that when compared to the large Childhood Development after Cochlear Implantation (CDaCI) Study sample of cochlear implant users, those from ethnic minorities had more delayed spoken language [81]; maternal education level—in general, better spoken language outcomes are observed in children whose mothers attained higher levels of education (e.g., [67,82,83]); gender—in general, girls achieved better language outcomes [82,84]; cognitive ability—as expected, children with higher cognitive abilities had better language outcomes [82,85,86]; ratings of parental sensitivity—children whose parents responded appropriately to their child’s communication attempts had better spoken language outcomes [66,87]; dynamics within the family itself—children whose families that reported lower levels of rigid behavioral control over their children, but higher levels of organization within the family itself, had better spoken language outcomes [88,89], and etiology—etiology works almost as a proxy for other factors (many identified here) in that the mechanism(s) through which the etiology of hearing loss influences hearing structures themselves, other systems, and development in general will influence outcomes (e.g., etiologies that influence the central nervous system more generally or that involve specific aspects of the central auditory system specifically, or that have associated cognitive delays/difficulties will result in poorer outcomes) [82,85,86]. Together, these factors only account for about half of the variability in spoken language outcomes. Keeping both the enormous individual differences and these predictive factors in mind, this section will summarize the average spoken language outcomes of pediatric cochlear implant recipients.

The average spoken language growth trajectories of children who receive cochlear implants dramatically change following cochlear implantation: spoken language growth is very slow prior to cochlear implantation regardless of the age at which the child receives the device, however, very quickly following cochlear implantation both average spoken language and speech perception trajectories improve (e.g., [52,66]). These positive post-operative growth trajectories are observed for both receptive and expressive language, as well as vocabulary. Additionally, children who receive their devices before 18 months of age have post-operative trajectories on average that begin to parallel the growth rates of hearing children (e.g., [52,66]), although they still lag behind in absolute language scores because they started behind when they received the device. Spoken language growth trajectories are shallower for children implanted through 36 to 48 months of age relative to those implanted before 18–24 months of age [52,66]. Together, these data reveal that the language gap between hearing children and DHH children implanted before approximately 18 months of age does not widen with development. In contrast, DHH children implanted after 18 months of age see language gaps that widen over time. This means that when implantation is delayed past approximately 18 months of age, not only are DHH children already far behind their hearing peers in language skills, their rate of spoken language acquisition is slower than their hearing peers, even with a cochlear implant. On average, the language gap on standardized measures is approximately 1 standard deviation (SD) below the mean [52,55,55,66,82,90,91). This 1-SD gap has remained relatively consistent for the last decade or so despite some of the most sophisticated signal processing to date. Again, these are simply averages, so educators will encounter children from across the spectrum: those who are scoring even higher than their age would suggest to those who struggle to even discriminate among words with different stress patterns, and thus, primarily use their device as an aid to speechreading or Total communication. This is why educators and intervention specialists have to be flexible with education and intervention plans for children with cochlear implants. Each child needs a truly individualized plan.

The vast majority of the literature on speech and language outcomes in children with cochlear implants uses standardized tests of spoken language. These tests have some advantages over non-standardized measures in that they allow the tester/researcher to compare the results to hearing children. Additionally, standardized spoken language tests are often used to determine if children receive services through the schools. These tests also have some disadvantages, though. They often do not test high-level language skills needed to develop deep peer relationships or to soar academically. Furthermore, they lack the ability to evaluate specific aspects of language development with which a particular child might struggle or specific aspects of language development particularly at-risk in children with hearing loss. Therefore, some investigators have argued that going beyond these standardized measures is important for quantifying their development across multiple domains of language [92], as well as capturing the full breadth of language development needed to optimize and provide efficient intervention [82].

Despite the ability to provide access to high-frequency cues not available in hearing aids, cochlear implants still lack the frequency resolution needed for perceiving fine frequency cues. Thus, children with cochlear implants display difficulties with morphological development, specifically those marking possessives, plurals, verb tense, and pronouns [93-96]. Additionally, children with cochlear implants show difficulties in syntactic development [82,94,96], correct use of verbs and adverbs [93,97], and prepositions [97]. This leads to children with cochlear implants having smaller lexicon sizes than hearing children [31,98]. Finally, children with cochlear implants tend to have shorter average utterance lengths than hearing children [99]. Across these different areas of language development, Nittrouer, Muir, Tietgens, Moberly, and Lowenstein [100] reported that through middle school, the types and magnitudes of deficits experienced by children with cochlear implants remain relatively consistent. Furthermore, there was a hierarchy of difficulty: children displayed the largest deficits with phonological skills, moderate deficits with lexical skills, and the least for morphosyntactic skills. Children with cochlear implants are particularly vulnerable to deficits in phonological development across childhood [101-103]. Importantly, acquiring literacy skills was strongly supported by phonological and lexical development—the two areas of greatest difficulty for children with cochlear implants [100].

Speech production of children with cochlear implants is also highly variable [104]. Articulation is often significantly impaired in children with cochlear implants [105], resulting in it being estimated that only about half of preschooler’s speech is intelligible [91]. Part of the reason for some children being difficult to understand, even those who have had their devices for an extended period of time, is that their phonetic inventories are not only missing sounds from their ambient language, but also contain sounds that do not appear in their ambient language [106]. Furthermore, there appears to be an effect of communication modality: inventories of oral communicators tend to contain more English segments than those of Total communicators. Conversely, non-English segments (such as uvular stops) appear more commonly in Total communicators’ than oral communicators’ inventories. This also extended to consonant clusters, in which oral communicators are more likely to successfully produce initial onset clusters correctly than Total communicators [107]. Finally, like hearing children much younger than themselves [108,109], children with cochlear implants tend to omit function words from their productions, thus producing more content than function words [110]. Word omission correlated with intelligibility. Together, these factors contribute to some DHH children’s difficulty in being understood.

## 11. Literacy Development in DHH Children Who Use Hearing Aids and Cochlear Implants

Similar to the outcomes in research on spoken language (and unsurprisingly), literacy outcomes in DHH children remain a significant area of risk despite recent efforts to emphasize literacy development and many changes in educational intervention [111]. There are conflicting reports in the literature regarding literacy achievement in DHH children. Some studies report that only a small fraction—approximately 10%—of graduating DHH high schoolers (of all linguistic backgrounds) read at grade level, and that the majority read at just the fourth-grade level, particularly as the degree of loss increases in severity (e.g., [112,113]). Other studies hold greater promise for more positive literacy prognoses. For example, in a large investigation of 181 children who received cochlear implants before age five years, Geers [114] showed that regardless of communication modality (oral or simultaneous/Total communication), just over half of the children had reading scores commensurate with their hearing peers. This proportion of children demonstrating literacy success is much higher than has been reported in other studies, certainly in earlier studies on cochlear implantation. To be certain, their success is due in part to their committed families, early interventionists, and their own hard work, but much of it is also due to children’s access to the ambient language around them and the component phonemes provided by the modern hearing technology that they have been fitted with at an early age that is simultaneously being conveyed through the orthography of the letters they are learning to decode and read as words, phrases, sentences, and passages. Despite the hopeful literacy gains made in the last decade or so by DHH children, achievement gaps between DHH children with hearing technology and hearing peers still persist (e.g., [44,114-117]). Moreover, as with spoken language outcomes, there is enormous variability in literacy achievement, with approximately half of DHH children with hearing technology achieving age-appropriate literacy skills, some approximately a year behind their hearing peers, and a smaller subset “exhibiting barely developed reading skills” in the studies with the more promising outcomes [114], (p. 66S). Typically, reading score gaps increase with age [118], although certain types of interventions have seen more positive outcomes using visual phonics-based instruction (e.g., [119-121]).

Literacy development relies on many skills and experiences. However, the two key building blocks are general language abilities (oral language and vocabulary) and phonological knowledge to break down printed words into parts, sometimes called phonological coding and awareness [116]. The role that each—top-down language and bottom-up phonological coding and awareness—plays in reading is hotly contested (e.g., [122]). DHH children have been used to test hypotheses about the role of each in literacy acquisition because they have language delays and deficits in phonological processing and encoding. They have also been studied because of the long-standing achievement gaps observed in their reading development.

Easterbrooks, Lederberg, Miller, Bergeron, and Connor [123], Nittrouer et al. [101,102], Nittrouer and Caldwell-Tarr [103], and others have demonstrated that DHH children with hearing technology have differing abilities to access the building blocks of reading. Factors that have been found to influence reading scores in DHH children with cochlear implants and hearing aids include: the educational environment (oral versus simultaneous communication in the classroom) [114,124,125], phonological awareness [114,122,126,127], speech intelligibility and language comprehension [114,126], and vocabulary and speechreading skills [128]. In a meta-analysis that examined DHH children’s reading scores but did not separate out hearing technology users from DHH children who used ASL and did not use hearing technology (a limitation of this meta-analysis), found that language accounted for most of the variability in reading scores across seven studies [129]. In a study that examined the contributions of many potential contributing factors, 72% of the variance in reading scores of children who received cochlear implants before age five years together was due to: age at onset of deafness, intelligence quotient, family socioeconomic status, gender, cochlear implant processing strategy, width of the dynamic range of the child’s MAP, working memory, phonological processing, speech production, and language abilities. In fact, together, language and speech production accounted for 45% of the variance in reading scores (note that speech perception was not significant) [114]. These results suggest that children’s oral language success contributed, as well as their speech intelligibility, almost half of the variability in reading scores of DHH children with cochlear implants. Moreover, phonological processing contributed additional variability, as did demographic factors. Results such as these support the tenet that phonological coding and awareness, as well as lexical knowledge, contribute heavily to reading ability, but so do a multitude of other factors that help support access to perceptual learning, attention, and general cognitive mechanisms that are important to becoming a competent reader.

Evidence from both hearing [129] and DHH populations [122] suggests that the development of phonemic awareness and reading are reciprocal or bidirectional processes that appear to support each other. In controlled studies across two different languages, children with hearing technology who received reading training showed enhanced phonological and morphological skill development [129,130]. These results suggest that the relationship between reading and phonological and morphological skill development is a complicated one that seems to work in an almost cyclical fashion that can feed off of itself

One method that has been effective in assisting DHH children in accessing the phonology of their auditory language and applying it to reading is an instruction method based in visual phonics [118-120]. This intervention method is exciting in part because the results show that it is beginning to close the achievement gap in reading for some DHH children with hearing technology [110-120], but also because of results like those of Kyle and Harris [131] that found that speechreading was the strongest single predictor of single-word reading ability, whereas vocabulary knowledge best predicted written sentence comprehension. The ability to use visual speech cues acts as a mediator for phonological awareness when auditory information is unreliable or inaccessible due to hearing loss, or as a supplement for all listeners under any situation, but especially under degraded listening situations. Combining the visual phonics with knowledge of the lexicon together contributes to the necessary decoding and comprehension necessary for reading.

Despite the strides that have been made in literacy research, significant unexplained variability and a large achievement gap remains for many DHH children. The majority of the data suggest that the language and literacy gaps are related to one another. That being said, some have argued that DHH children’s reading challenges might in fact *not* be reading-specific [132]. For example, Marschark and colleagues [133] have proposed that the reading difficulties experienced by DHH children might instead be a result of more language-general and cognitive factors—cognitive processing, language comprehension, and learning factors that contribute to reading. Certainly working memory and the phonological loop have been implicated in reading [134]. Further research is needed to better understand the role of these additional factors in literacy development. The differing views on the relative importance of top-down (language) and bottom-up (phonological coding and awareness) processes to reading for DHH children with cochlear implants and hearing aids is probably exacerbated by the variability in access to the LTASS experienced across children. A limitation of most of the investigations on this issue is that they: (1) do not describe the participant population in sufficient detail to glean their potential ability to use their hearing technology to develop phonemic coding and awareness competency; (2) do not separate out children in groups who are likely to differ in their language and phonological coding and awareness skills based on their auditory and audiovisual spoken language experience; and (3) do not include neurocognitive and sociodemographic factors known to influence language development in the analyses, which could indirectly (or perhaps directly) influence reading development. As more investigations converge on the sources of variability in reading outcomes, it will be exciting for new or modified evidence-based intervention strategies to be developed and applied to larger numbers of DHH children with hearing technology that is targeted to meet their individual needs.

## 12. Implications for Research

This review of current hearing technology for DHH children and its influence on spoken language and literacy development has highlighted some gaps in knowledge. Below are some of the most pressing research needs to fill those holes:

- Investigations into why clinical and laboratory results are so discrepant regarding children’s match-to-target of their hearing aid fittings.
- The first step in the marriage of hearing technology and spoken language development is making the LTASS fully audible. Access to high-quality auditory information is critical to spoken language development and an optimal hearing aid fitting that matches the prescribed gain target is step one in that process [50]. Over half of the time, clinical audiologists do *not* match the gain targets across frequency, meaning that the majority of children do not have optimal access to the LTASS [25,26]. This is a serious problem and one that needs to be addressed in research and training.
- The effects of digital noise reduction on speech perception, spoken language development, and learning environments in children who use hearing aids.
- The consequences of frequency lowering to speech perception and spoken language development, and its links to literacy in children who use hearing aids.
- The achievement gap between DHH and hearing children and relatedly, identifying sources of individual differences in spoken language and literacy outcomes in DHH children.
- Literacy intervention investigations that take into account individual differences of DHH children.
- Expanding the search for factors that influence literacy achievement in DHH children outside of traditional language and phonological awareness measures.
- At this time, approximately half of the variability in outcomes of DHH children has been identified, leaving much of the remaining variability unexplained.
- Identifying other sources of individual differences could lead to novel interventions for DHH children and their families, which could contribute to narrowing, or ideally closing, the achievement gap in spoken language and literacy.

## 13. Conclusions

Impressive advances in hearing technology have occurred in the last 30 years offering the opportunity for DHH children to have the adequate auditory access necessary to acquire spoken language with high-quality early intervention. While some children achieve outstanding spoken language and literacy outcomes, there remains a significant achievement gap between many DHH children and their hearing peers, even those who are identified early and receive appropriate early intervention with sophisticated technology. Addressing this achievement gap and identifying the sources of individual differences are two areas ripe for basic and translational research efforts. At present, we have a limited understanding of the development of DHH children in part because fields concerned with the development of DHH children have just begun to employ the widely-held view that human development is shaped by dynamic interactions between biology and environment [135]. This limitation contributes to intellectual isolation from other related scientific disciplines and thus neglects a key opportunity for understanding individual variability in pediatric DHH outcomes. There is a need for a comprehensive theoretical model that specifies factors that contribute to at-risk outcomes and their mechanisms of influence that can be empirically tested. Models that hold promise for this purpose are Biopsychosocial systems-based, because they incorporate the dynamic bidirectional relationships between systems that influence developmental trajectories [136,137]. A Systems approach recognizes that development does not occur in a vacuum [138], but rather emerges within rings of environmental influence from the level of the cell to proximal and distal rings of the environment. Our research group has proposed a Social-Behavioral Risk Model of development of DHH children with sensory aids in order to examine the role of family environment and family dynamics on spoken language and executive function outcomes to begin capturing novel sources of variability in spoken language outcomes [139]. Kronenberger and Pisoni [140] have proposed the Auditory Neurocognitive Model to explain neurocognitive outcomes in DHH children with cochlear implants. Both of these models apply Biopsychosocial systems theory to account for the complex, dynamic, and reciprocal interactions and influences of factors on outcomes in DHH children that occur at neurobiological, cognitive, and psychosocial levels. The marrying of sophisticated hearing technology, processing by the brain, and a fuller, deeper understanding of the complex environmental and biological factors that shape development will help to maximize spoken language outcomes in DHH children and contribute to laying the groundwork for successful literacy and academic outcomes, particularly for the next generation of pediatric hearing aid and cochlear implant users.
